# Breast Mass and Lytic Bone Lesions: A Rare Presentation of Non-Hodgkin's Lymphoma Arising in the Breast

**DOI:** 10.1155/2013/547171

**Published:** 2013-10-21

**Authors:** Hira Ali, Oladapo Yeku, Daniel Giesler, Ryan Campbell-Massa, Faye Gao, Ali Imran Amjad

**Affiliations:** ^1^Division of General Internal Medicine, Department of Medicine, University of Pittsburgh Medical Centre, Pittsburgh, PA 15213, USA; ^2^Division of Hematopathology, Department of Pathology, University of Pittsburgh Medical Centre, Pittsburgh, PA 15213, USA; ^3^Division of Hematology/Oncology, Department of Medicine, University of Pittsburgh Medical Centre, Pittsburgh, PA 15232, USA

## Abstract

*Background*. Primary breast lymphoma is a rare malignancy representing less than 1% of all tumors presenting in the breast. *Case Presentation*. A 55-year-old woman presented with altered mental status due to severe hypercalcemia and was found to have a large breast mass with lytic bone lesions in the calvarium of the skull. Biopsy of the mass revealed diffuse large B-cell lymphoma. Staging workup did not reveal any visceral organ or distant lymph node involvement. The patient's bone marrow biopsy was positive for involvement with lymphoma. Interestingly, the patient also had a non quantifiable IgA kappa monoclonal protein in the serum. *Conclusion*. Here, we describe a common presentation in medical oncology, that is, a patient presenting as a clinically advanced breast tumor with hypercalcemia from lytic bone lesions. However, diagnostic workup led to the diagnosis of another common malignancy in an uncommon location, namely, diffuse large B-cell lymphoma arising in the breast.

## 1. Case Report

A 55-year-old Caucasian female with infrequent medical followup and no comorbidities was sent via ambulance to the hospital when coworkers noticed she was confused and not behaving her usual self. On interview, the patient thought she might have been dehydrated and reported recent increase in urinary frequency. She also reported a lump in her left breast which she first noticed six months ago and had been progressively getting larger. Her prior history included a remote retinal tear, and she took no medications. She was an active smoker for 30 years and her family history was negative for breast cancer or any other form of malignancy. Review of systems was positive for some lethargy and intermittent back pain. She denied fever, chills, night sweats, weight loss, headaches, focal weakness, abdominal pain, nausea, vomiting, diarrhea, cough, chest pain, shortness of breath, dysuria, or hematuria. Physical exam was pertinent for a firm, nontender mass in the left breast measuring approximately 15 cm in size with extension into the axillary region. There were no skin changes, nipple discharge, or signs of inflammation. Notably there was no lymphadenopathy or hepatosplenomegaly appreciated.

**Figure 1:**
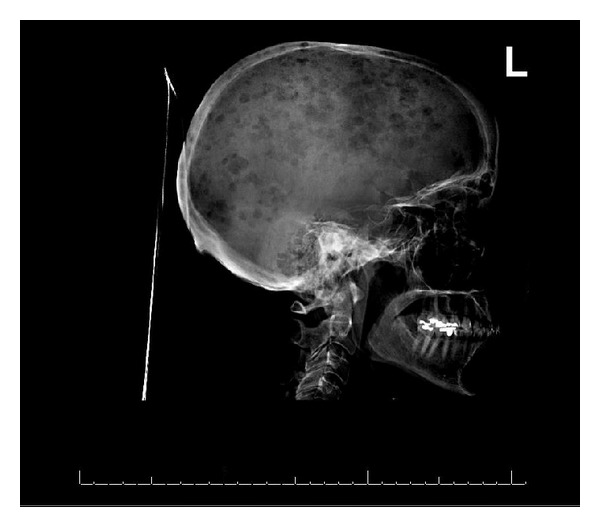
X-ray of the head showing multiple lucent calvarial defects described as lytic lesions.

Initial serum chemistries showed multiple metabolic derangements including acute kidney injury (Cr 2.1 mg/dL), profound hypercalcemia (adjusted serum calcium 20.3 mg/dL) and hyperuricemia (uric acid 11.1 mg/dL) (Table 1). CT scan of the head showed no parenchymal abnormalities but revealed multiple lucent calvarial defects consistent with lytic lesions which were also seen on a plain film (Figure 1).

**Figure 2:**
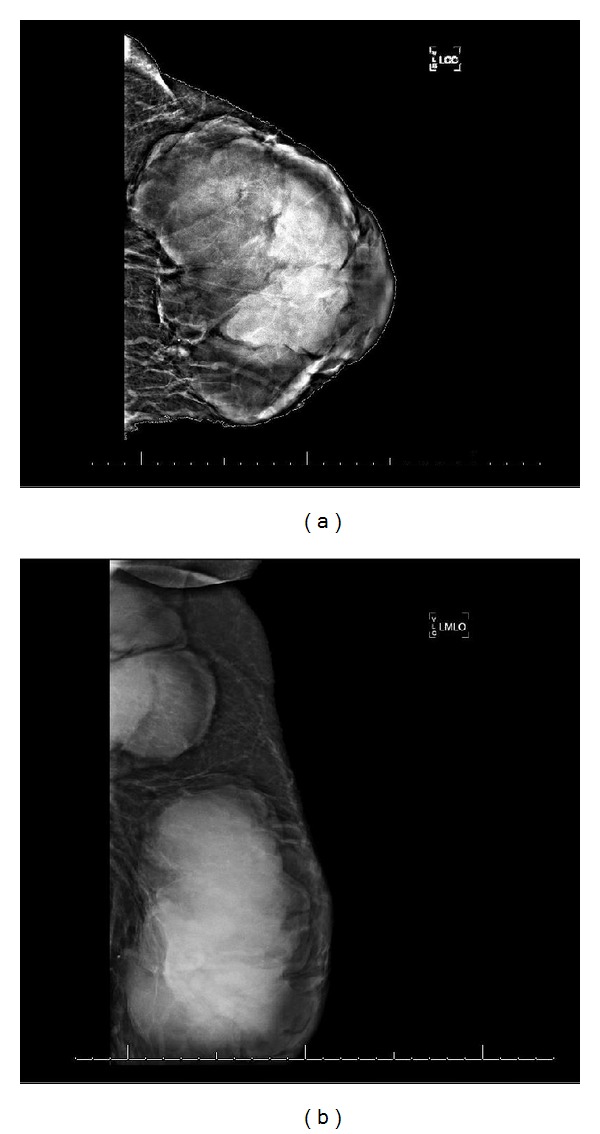
Breast Mammogram. (a) A large, hyperdense lobular shaped mass with smooth margins in the left breast, measuring 19 × 14 × 17 cm. (b) Left axilla showing a 6 cm large abnormal lymph node mass.

**Figure 3:**
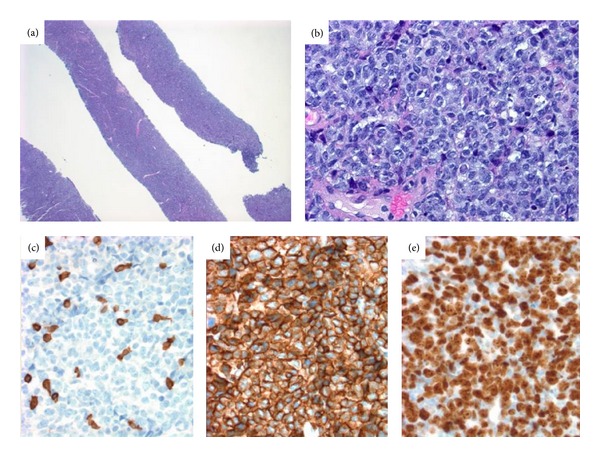
Ultrasound guided core biopsy. (a) Hematoxylin and eosin-stained section shows core needle core biopsy with dense infiltrate of atypical lymphocytes with no residual normal breast tissue being identified (20x). (b) High magnification to demonstrate sheets of large transformed cells with round to irregular nuclei, dispersed chromatin, and prominent nucleoli (400x). Immunohistochemistry stains for (c) CD3; (d) CD20; (e) Ki-67.

Mammogram confirmed a large, hyperdense lobular shaped mass with smooth margins in the left breast, measuring 19 × 14 × 17 cm. There was also a 6 cm abnormal lymph node in the left axilla (Figures 2(a) and 2(b)). Ultrasound-guided core needle biopsy of the breast mass revealed dense infiltrate of atypical lymphocytes, and no residual normal breast tissue was identified. The histologic sections demonstrated sheets of large transformed cells with round to irregular nuclei, dispersed chromatin, prominent nucleoli, and many mitoses. There was prominent apoptotic material, focal areas of geographic necrosis, and some areas with a “starry sky appearance.” Immunostains were strongly positive for CD20, CD3, and high Ki67 index (Figure 3). Fluorescent in-situ hybridization (FISH) performed on this biopsy specimen was positive for bcl-6 gene rearrangement. In-situ hybridization stain for Epstein Barr virus was negative. Pathological diagnosis was diffuse large B-cell lymphoma, not otherwise specified, with some high-grade features and a non germinal center phenotype. Fine needle aspiration biopsy of left axillary lymph node also revealed atypical cells, favoring lymphoma. CT chest, abdomen, and pelvis was negative for visceral or lymph node involvement.

Bone marrow biopsy performed to complete the staging workup showed marrow infiltration with CD20^+^ B-cell lymphoma comprising 25% of marrow cellularity. Interestingly there was an increase in plasma cell cellularity (7% by manual differential) and flow cytometry showed an abnormal phenotype (CD19^−^, CD56^+^, and kappa-light chain restricted). Finally cytogenetic analysis showed multiple chromosomal abnormalities (48,X,i(X)(p10), del(9)(p21p23), +12, add(14)(q32), add(17)(q25), +18[4]/46, XX[16]nuc ish(BCL6x2)).

Serum protein electrophoresis (SPEP) showed a non quantifiable IgA kappa monoclonal protein on immunofixation. Urine protein electrophoresis (UPEP) was negative for monoclonal protein. A skeletal survey was done and lytic lesions were found only in the calvarium of the skull (Figure 1).

The patient was started on intravenous fluids, calcitonin, and bisphosphonates for hypercalcemia. Rasburicase was given for hyperuricemia. Her kidney function and electrolyte abnormalities normalized within 48 hours of hospitalization and altered mental status resolved. After staging workup, the patient was given first cycle of R-CHOP chemotherapy with rituximab, cyclophosphamide, doxorubicin, vincristine, and prednisone. She had no evidence of tumor lysis and was discharged with appropriate followup to complete her subsequent cycles of chemotherapy.

## 2. Discussion

Primary breast lymphoma is a rare malignancy. First being described in 1959, it currently represents less than 1% of all breast tumors. Evidence regarding initial presentation, clinicopathologic features, natural history, and response to treatment comes primarily from retrospective studies and case series [1–4]. One such report was based on a large single center study from France evaluating 45 patients seen between 1986 and 2004. In that study, diffuse large B-cell constituted the predominant histology in 83% of those cases [2]. Bone marrow involvement is present in 13% of all cases of primary breast lymphoma. The patient described in this report was classified as Ann Arbor Stage IV with extra lymphatic involvement, lytic bone lesions, and a positive bone marrow biopsy. Revised International Prognostication Index, (Revised IPI Score) was classified as poor risk (3 of 5 factors, namely Stage IV disease, elevated LDH, and more than >1 extranodal site). What was more peculiar about the case was the unusual initial presentation with severe hypercalcemia and lytic bone lesions. Lymphomas are not typically associated with lytic bone lesions, and this case may be the first ever report of such occurrence with a primary beast lymphoma. Lytic bone lesions are a common manifestation of the usual variety of advanced metastatic breast cancer and are also frequently seen with multiple myeloma. Given the appearance of plain radiograph of the skull (Figure 1), workup was pursued on the lines of multiple myeloma but was non diagnostic. The composite of clinicopathologic/radiological findings in this case, that is, lytic bone lesions, non quantifiable monoclonal paraproteinemia, and slight bone marrow plasma cell predominance, may indicate monoclonal gammopathy of undetermined significance. Conversely, this could also represent a plasma cell dyscrasia with concomitant non-Hodgkin's lymphoma. Anecdotally, monoclonal gammopathy has been described in association with lymphomas (incidence 5–81%) [5]. However the exact incidence and clinical significance remains unclear.

## 3. Conclusion

This paper describes a common clinical presentation of a breast mass and lytic bone lesions. However, workup revealed an extremely rare presentation of primary non-Hodgkin's lymphoma involving the breast. Of interest there was concomitant weak paraproteinemia, without evidence of multiple myeloma.

## Figures and Tables

**Table 1:** Laboratory values at presentation and discharge with UPMC reference values.

Laboratory	Admission	Discharge	Reference value
Na	136	141	136–146 mMol/L
K	2.9	3.2	3.5–5.0 mMol/L
Cl	95	98	98–107 mMol/L
CO_2_	30	30	21–31 mMol/L
Bun	34	15	8–26 mg/dL
Cr	2.1	0.6	0.5–1.4 mg/dL
Ca (adjusted)	20.3	6.8	8.4–10.2 mg/dL
Ca (ionized)	2.04	1.11	1.15–1.29 mMol/L
Mg	1.2	1.6	1.6–2.3 mg/dL
Phos	2.8	3.1	2.5–4.6 mg/dL
Albumin	2.7	2.4	3.4–5 gm/dL
Total protein	8.0	5.3	6.3–7.7 gm/dL
Uric acid	11.1	3.5	2.5–7.5 mg/dL
LDH	268	552	<171 IU/L
WBC	8.6	8.1	3.8–10.6 × 10*E* + 9/L
Hgb	11	8.1	11.6–14.6 gm/dL
Hct	31.8	23.9	34.1–43.3%
Platelets	290	235	156–369 × 10*E* + 09/L
